# Supplementary figures and images for: Design of an enhanced feature point matching algorithm utilizing 3D laser scanning technology for sculpture design
Source: PeerJ Comput Sci. 2025 Jan 3;11:e2628. doi: 10.7717/peerj-cs.2628 (PMC11784803; doi:10.7717/peerj-cs.2628)

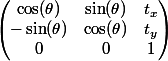

Supplement: Supplemental Information 1 [file peerj-cs-11-2628-s001.zip › code/doc/images/2dTransMatrix.gif]

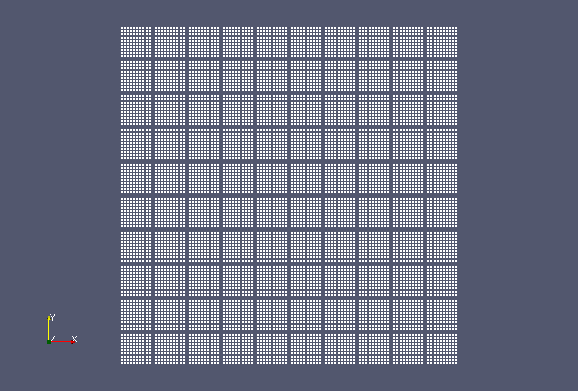

Supplement: Supplemental Information 1 [file peerj-cs-11-2628-s001.zip › code/doc/images/2dtestgrid.png]

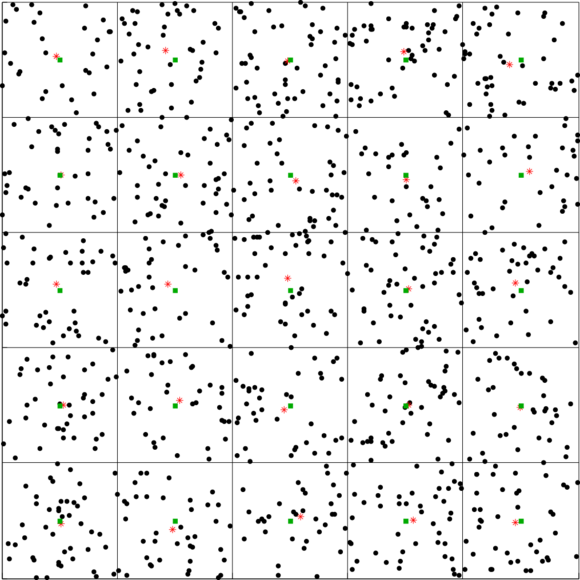

Supplement: Supplemental Information 1 [file peerj-cs-11-2628-s001.zip › code/doc/images/2dvxgrid.png]

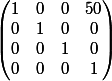

Supplement: Supplemental Information 1 [file peerj-cs-11-2628-s001.zip › code/doc/images/3d50mTrans.gif]

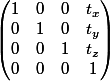

Supplement: Supplemental Information 1 [file peerj-cs-11-2628-s001.zip › code/doc/images/3dTransMatrix.gif]

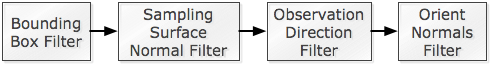

Supplement: Supplemental Information 1 [file peerj-cs-11-2628-s001.zip › code/doc/images/DefaultConvertChain.png]

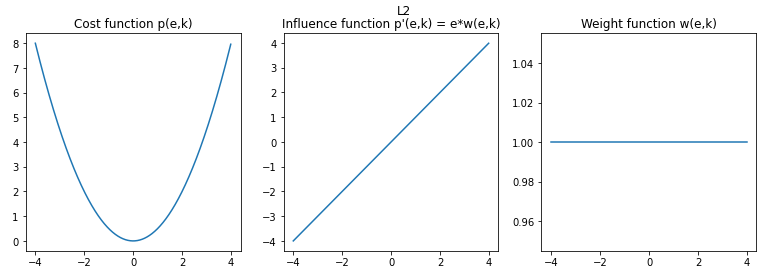

Supplement: Supplemental Information 1 [file peerj-cs-11-2628-s001.zip › code/doc/images/L2_nok.png]

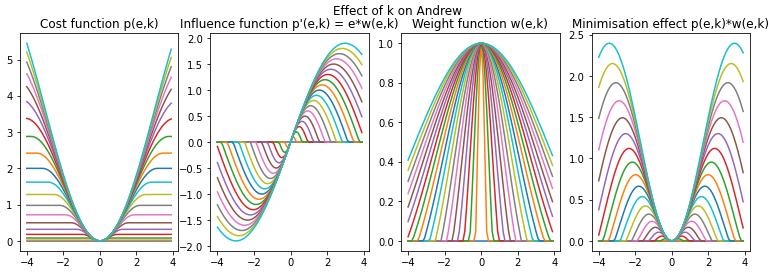

Supplement: Supplemental Information 1 [file peerj-cs-11-2628-s001.zip › code/doc/images/andrew_multik.png]

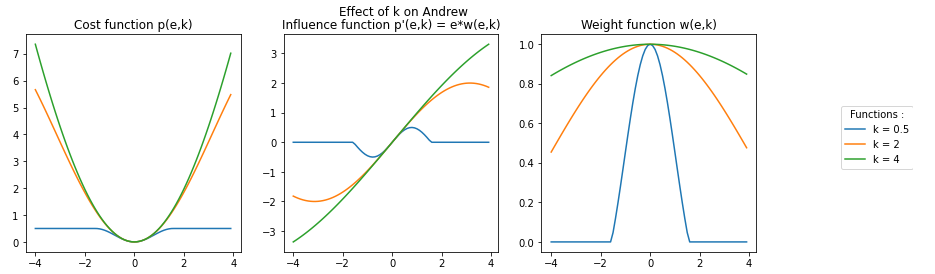

Supplement: Supplemental Information 1 [file peerj-cs-11-2628-s001.zip › code/doc/images/andrew_threek.png]

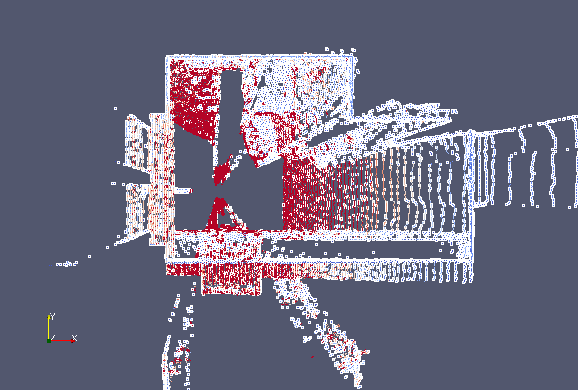

Supplement: Supplemental Information 1 [file peerj-cs-11-2628-s001.zip › code/doc/images/appt_0_maxdens.png]

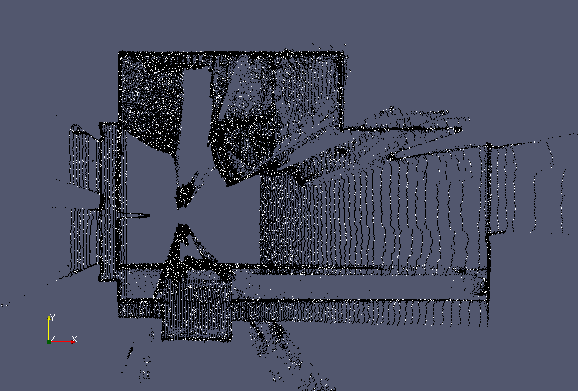

Supplement: Supplemental Information 1 [file peerj-cs-11-2628-s001.zip › code/doc/images/appt_0_rand.png]

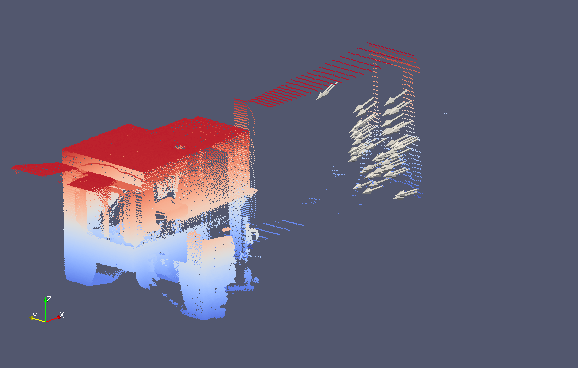

Supplement: Supplemental Information 1 [file peerj-cs-11-2628-s001.zip › code/doc/images/appt_dir.png]

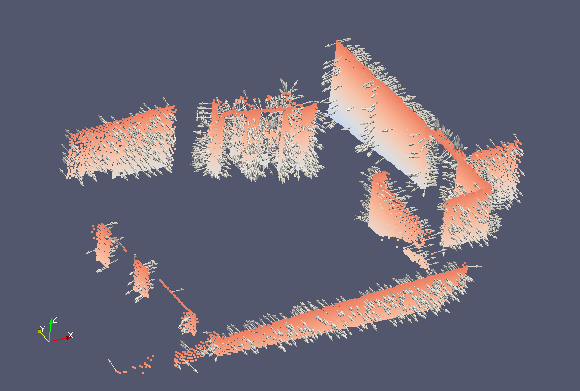

Supplement: Supplemental Information 1 [file peerj-cs-11-2628-s001.zip › code/doc/images/appt_norm.png]

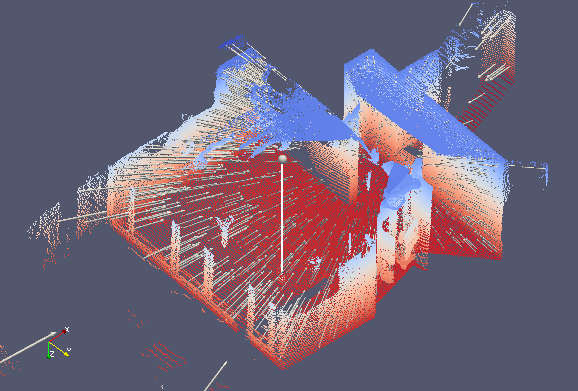

Supplement: Supplemental Information 1 [file peerj-cs-11-2628-s001.zip › code/doc/images/appt_obs_dir.png]

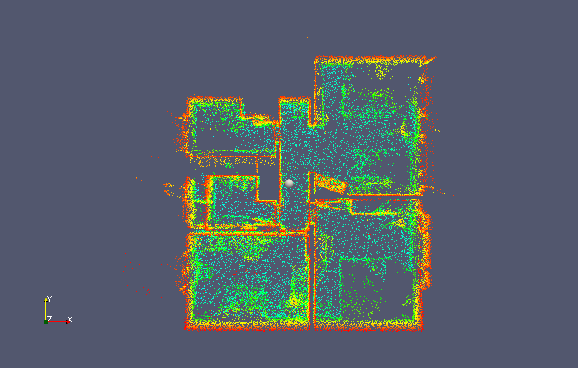

Supplement: Supplemental Information 1 [file peerj-cs-11-2628-s001.zip › code/doc/images/appt_rand.png]

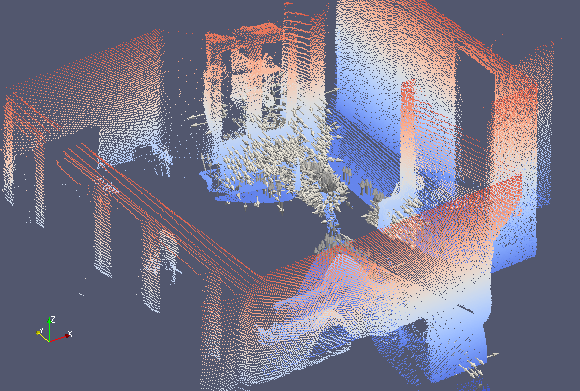

Supplement: Supplemental Information 1 [file peerj-cs-11-2628-s001.zip › code/doc/images/appt_samp_norm_dense.png]

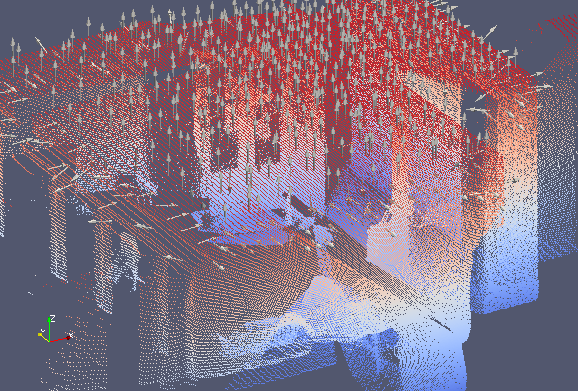

Supplement: Supplemental Information 1 [file peerj-cs-11-2628-s001.zip › code/doc/images/appt_samp_norm_sparse.png]

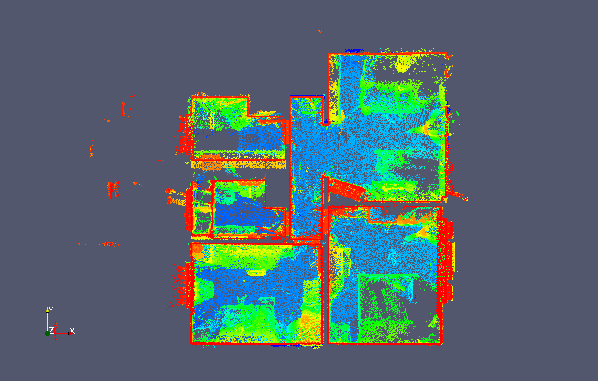

Supplement: Supplemental Information 1 [file peerj-cs-11-2628-s001.zip › code/doc/images/appt_top.png]

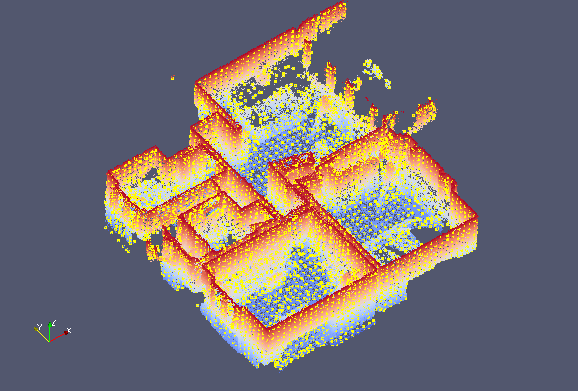

Supplement: Supplemental Information 1 [file peerj-cs-11-2628-s001.zip › code/doc/images/appt_voxel.png]

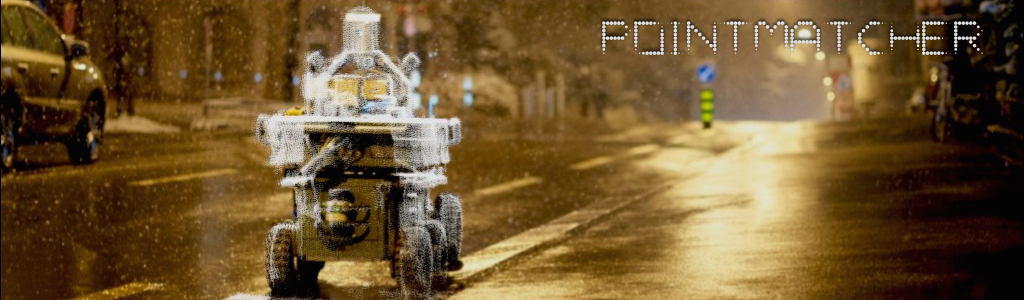

Supplement: Supplemental Information 1 [file peerj-cs-11-2628-s001.zip › code/doc/images/banner_dark.jpeg]

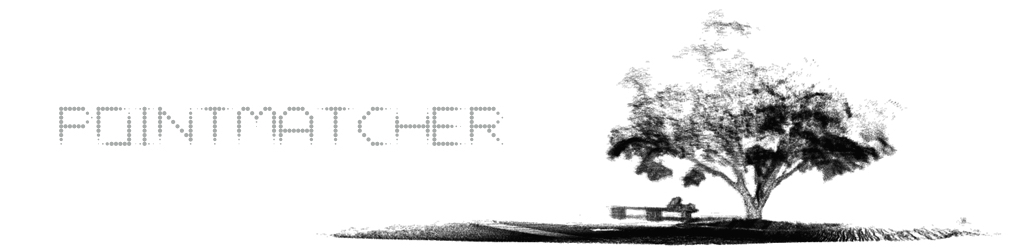

Supplement: Supplemental Information 1 [file peerj-cs-11-2628-s001.zip › code/doc/images/banner_light.jpeg]

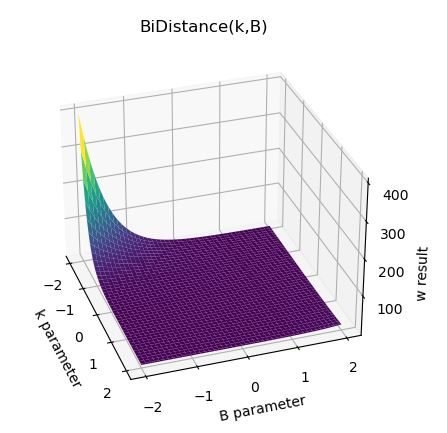

Supplement: Supplemental Information 1 [file peerj-cs-11-2628-s001.zip › code/doc/images/bidistance_multikB.jpg]

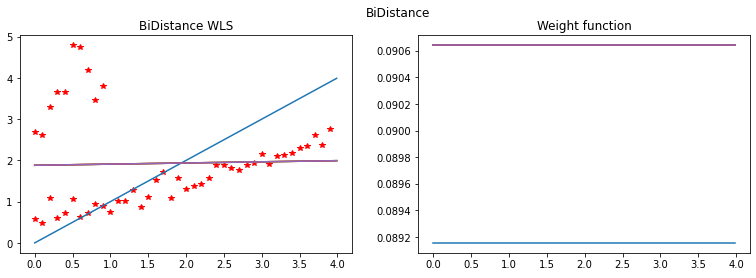

Supplement: Supplemental Information 1 [file peerj-cs-11-2628-s001.zip › code/doc/images/bidistance_nok.png]

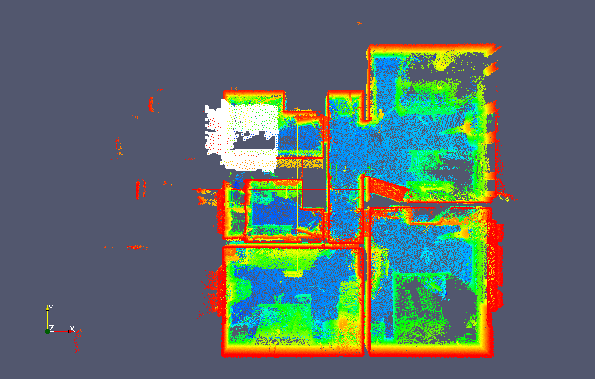

Supplement: Supplemental Information 1 [file peerj-cs-11-2628-s001.zip › code/doc/images/box_filt.png]

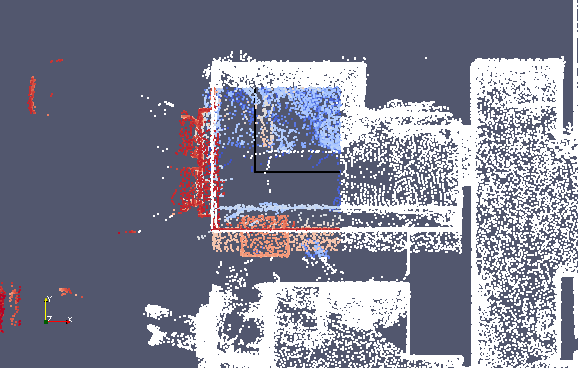

Supplement: Supplemental Information 1 [file peerj-cs-11-2628-s001.zip › code/doc/images/box_filt_inside.png]

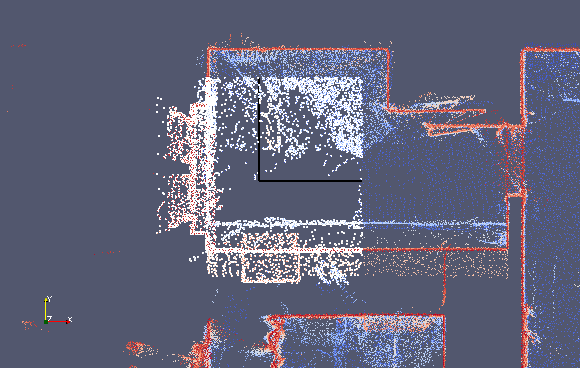

Supplement: Supplemental Information 1 [file peerj-cs-11-2628-s001.zip › code/doc/images/box_filt_outside.png]

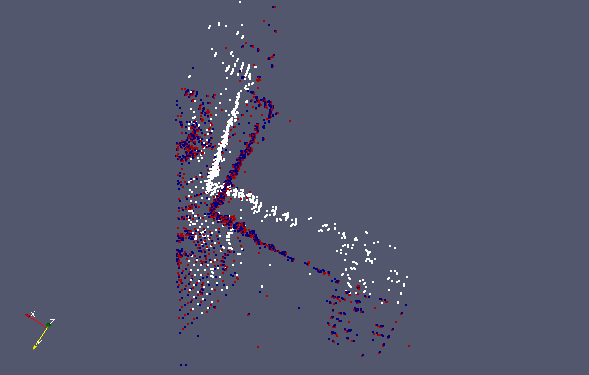

Supplement: Supplemental Information 1 [file peerj-cs-11-2628-s001.zip › code/doc/images/car_example.png]

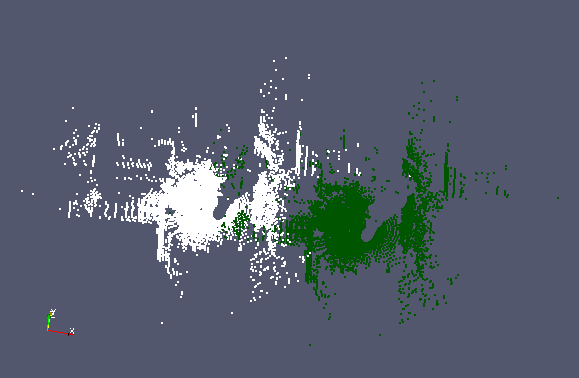

Supplement: Supplemental Information 1 [file peerj-cs-11-2628-s001.zip › code/doc/images/car_translated.png]

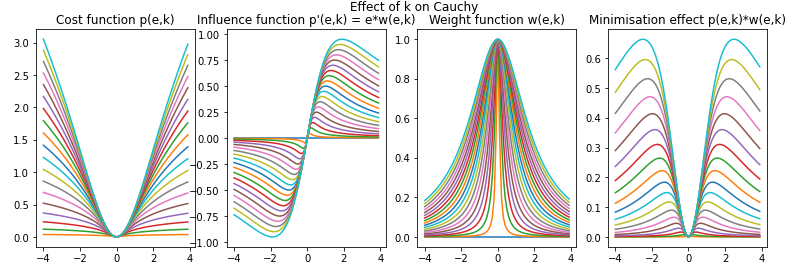

Supplement: Supplemental Information 1 [file peerj-cs-11-2628-s001.zip › code/doc/images/cauchy_multik.png]

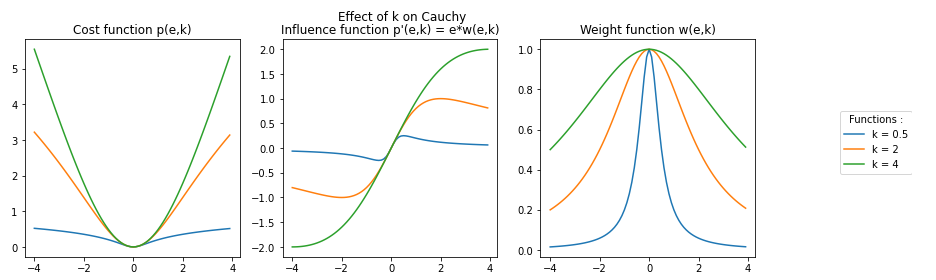

Supplement: Supplemental Information 1 [file peerj-cs-11-2628-s001.zip › code/doc/images/cauchy_threek.png]

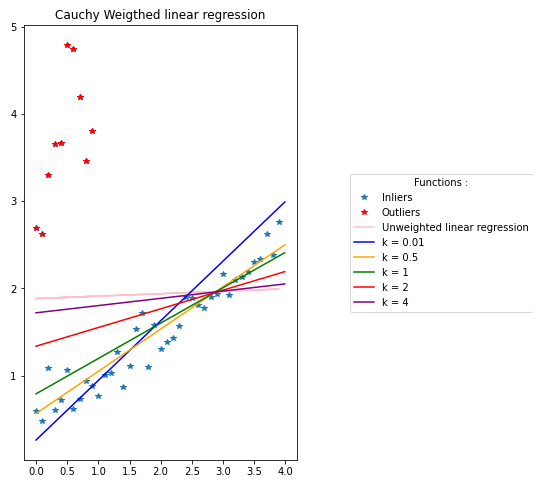

Supplement: Supplemental Information 1 [file peerj-cs-11-2628-s001.zip › code/doc/images/cauchy_weighted_linear_regression.png]

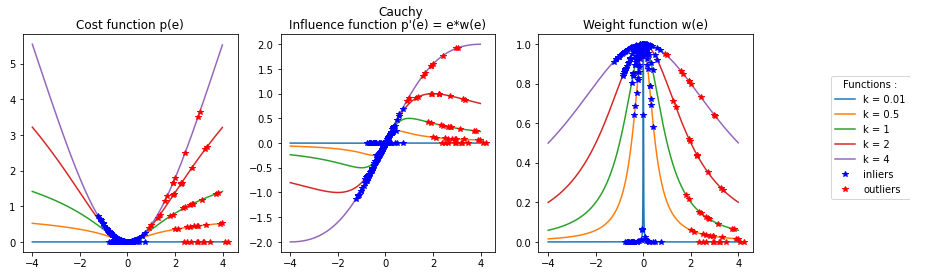

Supplement: Supplemental Information 1 [file peerj-cs-11-2628-s001.zip › code/doc/images/cauchy_with_points.png]

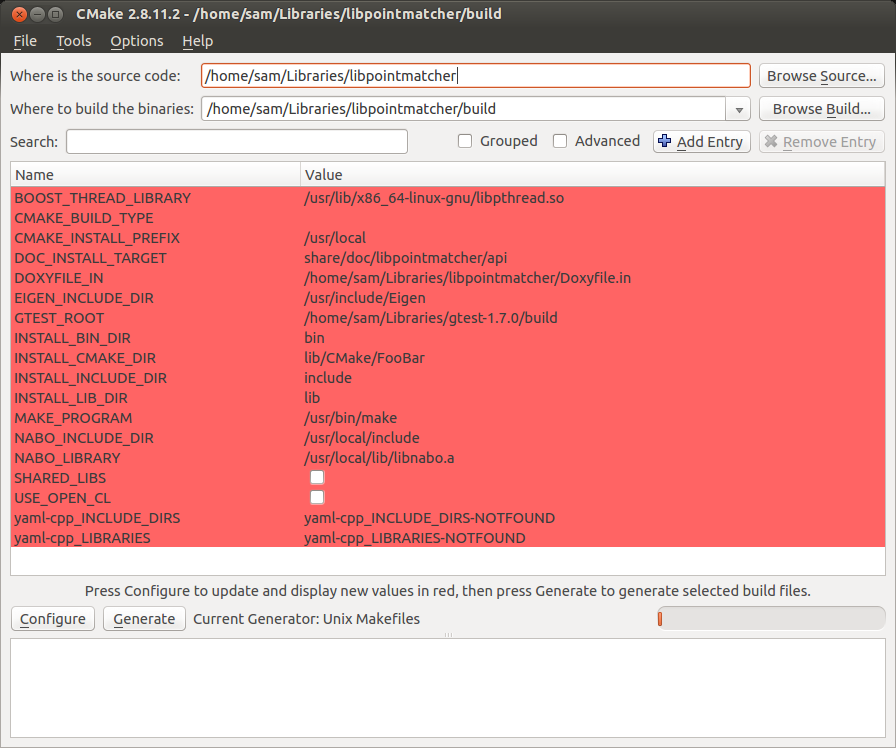

Supplement: Supplemental Information 1 [file peerj-cs-11-2628-s001.zip › code/doc/images/cmake_screenshot.png]

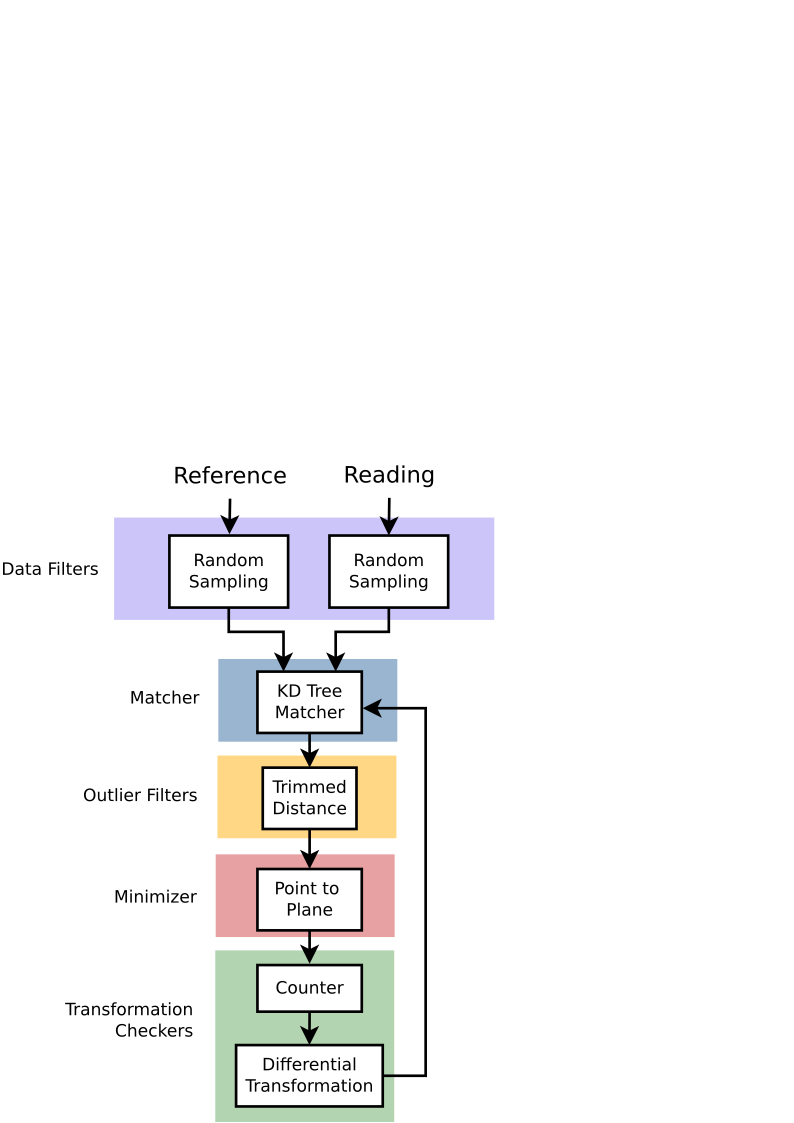

Supplement: Supplemental Information 1 [file peerj-cs-11-2628-s001.zip › code/doc/images/default_icp_chain.png]

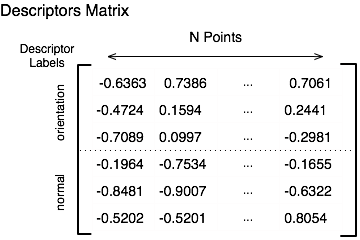

Supplement: Supplemental Information 1 [file peerj-cs-11-2628-s001.zip › code/doc/images/descriptorsMatrix.png]

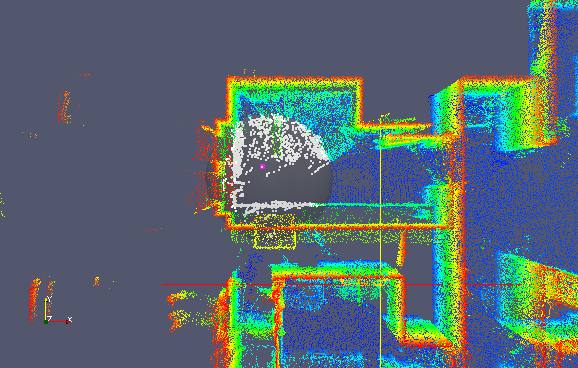

Supplement: Supplemental Information 1 [file peerj-cs-11-2628-s001.zip › code/doc/images/distance_limit.png]

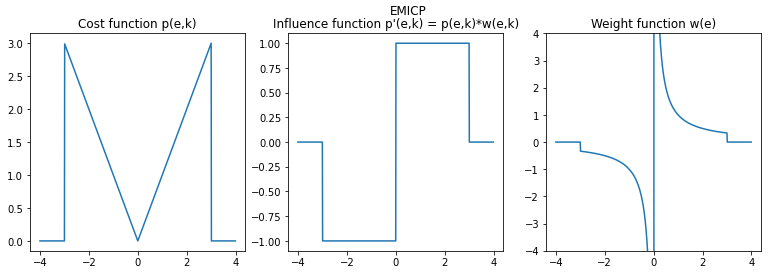

Supplement: Supplemental Information 1 [file peerj-cs-11-2628-s001.zip › code/doc/images/emicp_nok.png]

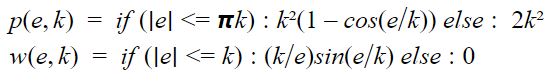

Supplement: Supplemental Information 1 [file peerj-cs-11-2628-s001.zip › code/doc/images/eq_andrew.JPG]

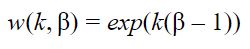

Supplement: Supplemental Information 1 [file peerj-cs-11-2628-s001.zip › code/doc/images/eq_bidistance.JPG]

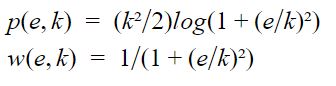

Supplement: Supplemental Information 1 [file peerj-cs-11-2628-s001.zip › code/doc/images/eq_cauchy.JPG]

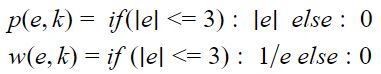

Supplement: Supplemental Information 1 [file peerj-cs-11-2628-s001.zip › code/doc/images/eq_emicp.JPG]

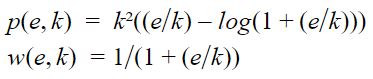

Supplement: Supplemental Information 1 [file peerj-cs-11-2628-s001.zip › code/doc/images/eq_fair.JPG]

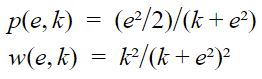

Supplement: Supplemental Information 1 [file peerj-cs-11-2628-s001.zip › code/doc/images/eq_fgr.JPG]

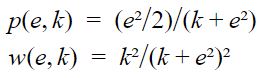

Supplement: Supplemental Information 1 [file peerj-cs-11-2628-s001.zip › code/doc/images/eq_geman.JPG]

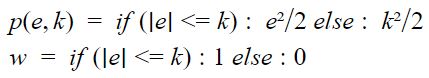

Supplement: Supplemental Information 1 [file peerj-cs-11-2628-s001.zip › code/doc/images/eq_gicp.JPG]

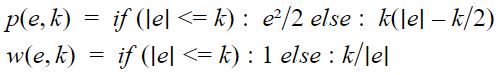

Supplement: Supplemental Information 1 [file peerj-cs-11-2628-s001.zip › code/doc/images/eq_huber.JPG]

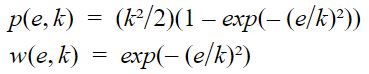

Supplement: Supplemental Information 1 [file peerj-cs-11-2628-s001.zip › code/doc/images/eq_kc.JPG]

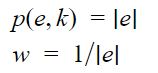

Supplement: Supplemental Information 1 [file peerj-cs-11-2628-s001.zip › code/doc/images/eq_l1.JPG]

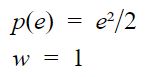

Supplement: Supplemental Information 1 [file peerj-cs-11-2628-s001.zip › code/doc/images/eq_l2.JPG]

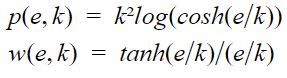

Supplement: Supplemental Information 1 [file peerj-cs-11-2628-s001.zip › code/doc/images/eq_logistic.JPG]

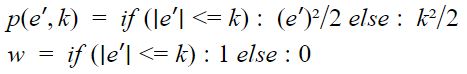

Supplement: Supplemental Information 1 [file peerj-cs-11-2628-s001.zip › code/doc/images/eq_maxdist.JPG]

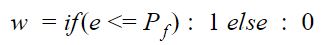

Supplement: Supplemental Information 1 [file peerj-cs-11-2628-s001.zip › code/doc/images/eq_percentile.JPG]

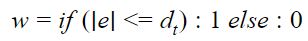

Supplement: Supplemental Information 1 [file peerj-cs-11-2628-s001.zip › code/doc/images/eq_rmt.JPG]

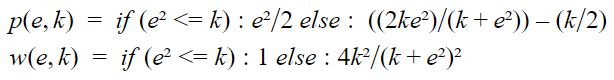

Supplement: Supplemental Information 1 [file peerj-cs-11-2628-s001.zip › code/doc/images/eq_sc.JPG]

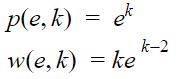

Supplement: Supplemental Information 1 [file peerj-cs-11-2628-s001.zip › code/doc/images/eq_sparse.JPG]

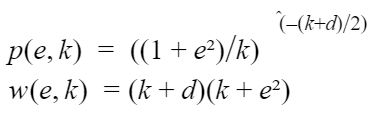

Supplement: Supplemental Information 1 [file peerj-cs-11-2628-s001.zip › code/doc/images/eq_student.JPG]

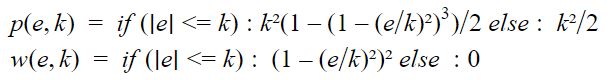

Supplement: Supplemental Information 1 [file peerj-cs-11-2628-s001.zip › code/doc/images/eq_tukey.JPG]

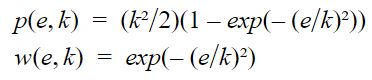

Supplement: Supplemental Information 1 [file peerj-cs-11-2628-s001.zip › code/doc/images/eq_welsch.JPG]

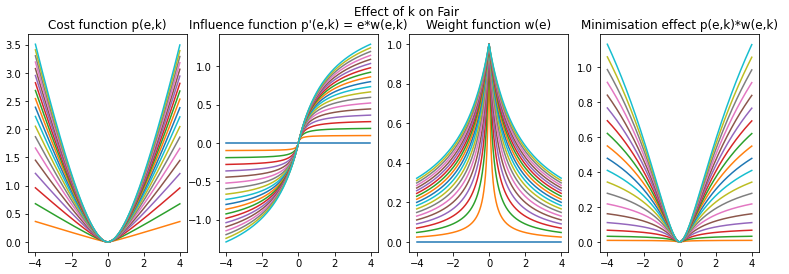

Supplement: Supplemental Information 1 [file peerj-cs-11-2628-s001.zip › code/doc/images/fair_multik.png]

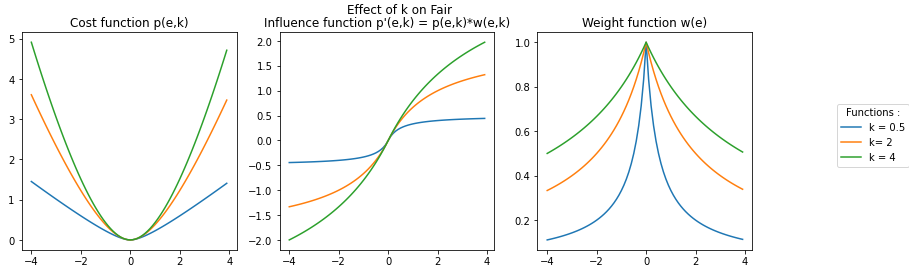

Supplement: Supplemental Information 1 [file peerj-cs-11-2628-s001.zip › code/doc/images/fair_threek.png]

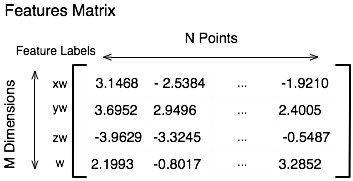

Supplement: Supplemental Information 1 [file peerj-cs-11-2628-s001.zip › code/doc/images/featuresMatrix.png]

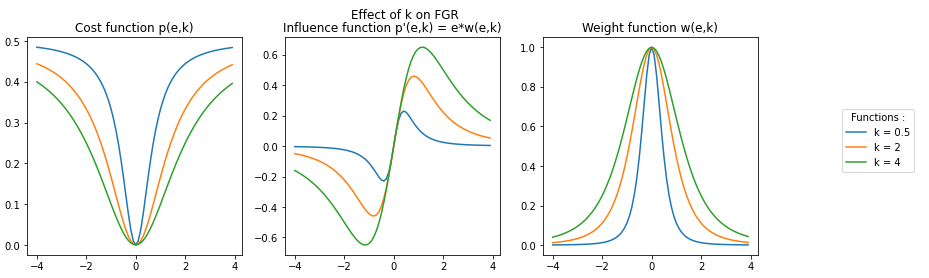

Supplement: Supplemental Information 1 [file peerj-cs-11-2628-s001.zip › code/doc/images/fgr.png]

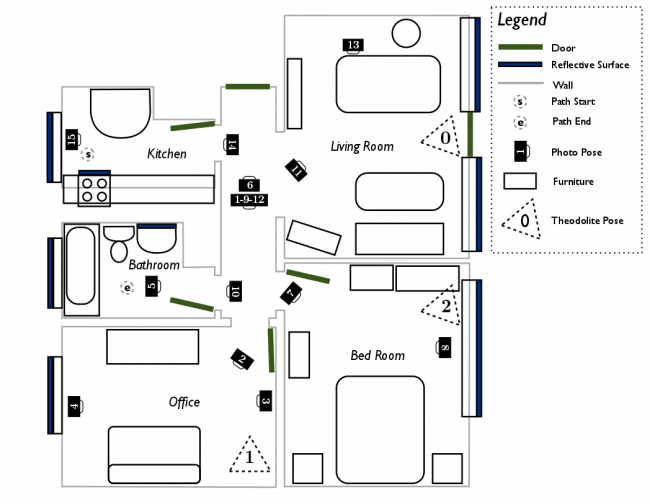

Supplement: Supplemental Information 1 [file peerj-cs-11-2628-s001.zip › code/doc/images/floor_plan.png]

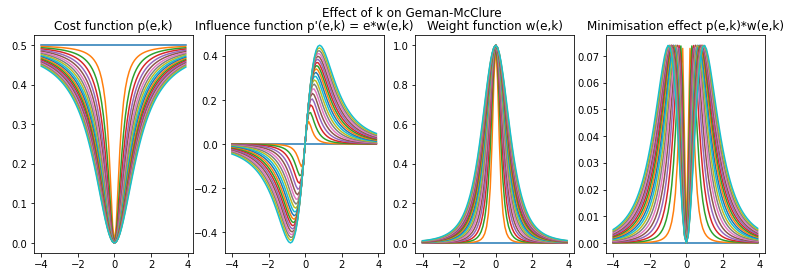

Supplement: Supplemental Information 1 [file peerj-cs-11-2628-s001.zip › code/doc/images/geman_multik.png]

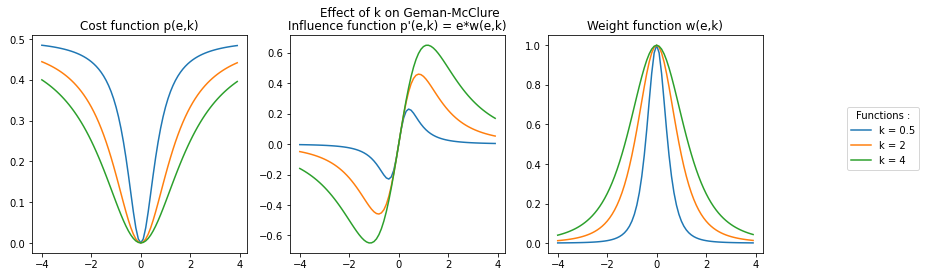

Supplement: Supplemental Information 1 [file peerj-cs-11-2628-s001.zip › code/doc/images/geman_threek.png]

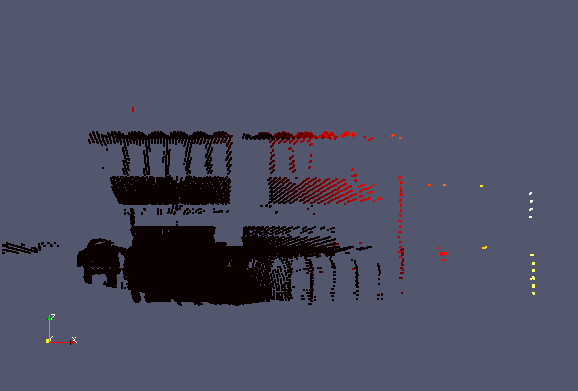

Supplement: Supplemental Information 1 [file peerj-cs-11-2628-s001.zip › code/doc/images/hg_noise.png]

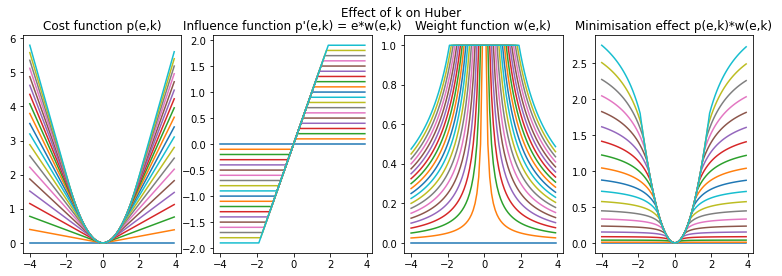

Supplement: Supplemental Information 1 [file peerj-cs-11-2628-s001.zip › code/doc/images/huber_multik.png]

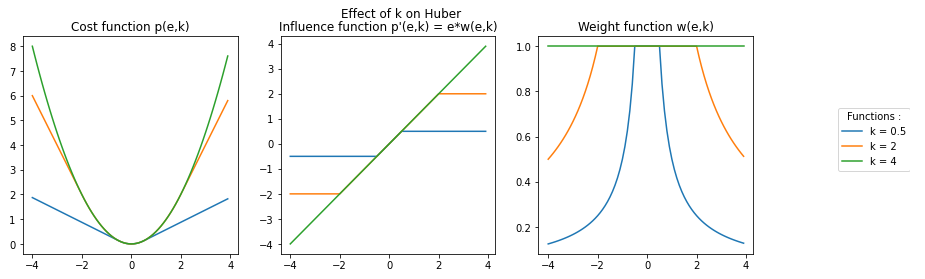

Supplement: Supplemental Information 1 [file peerj-cs-11-2628-s001.zip › code/doc/images/huber_threek.png]

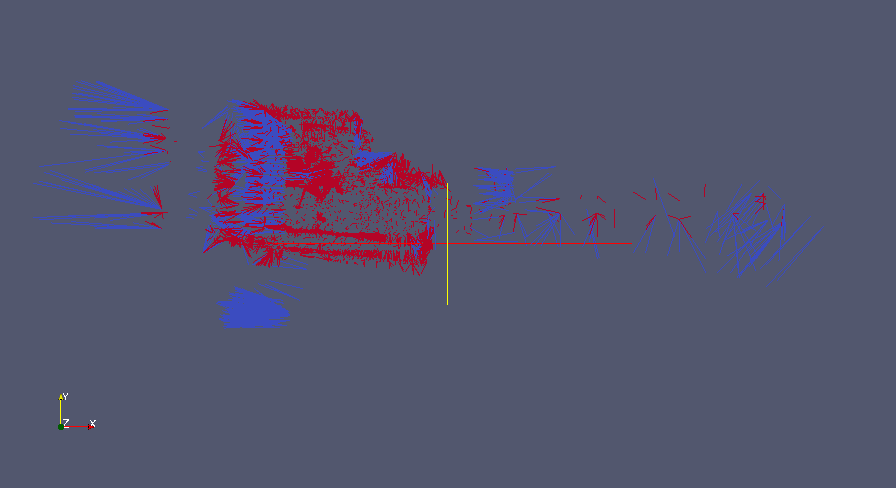

Supplement: Supplemental Information 1 [file peerj-cs-11-2628-s001.zip › code/doc/images/icp_tutorial_links.gif]

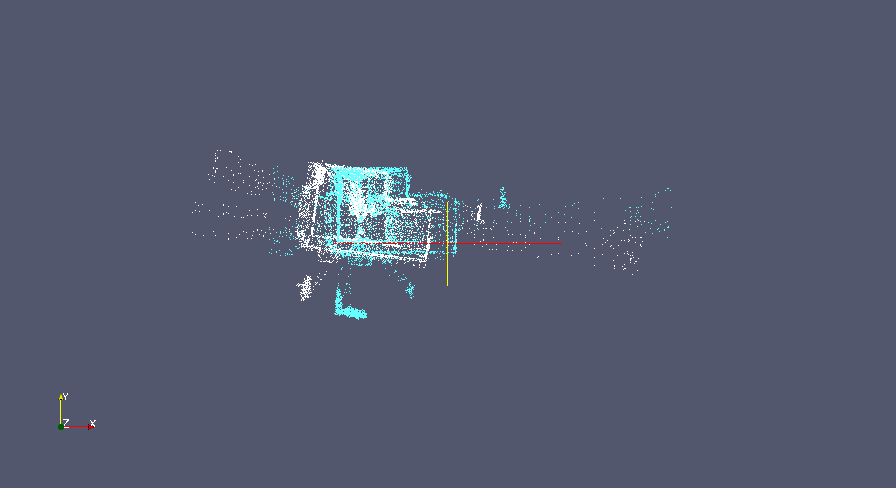

Supplement: Supplemental Information 1 [file peerj-cs-11-2628-s001.zip › code/doc/images/icp_tutorial_reading.gif]

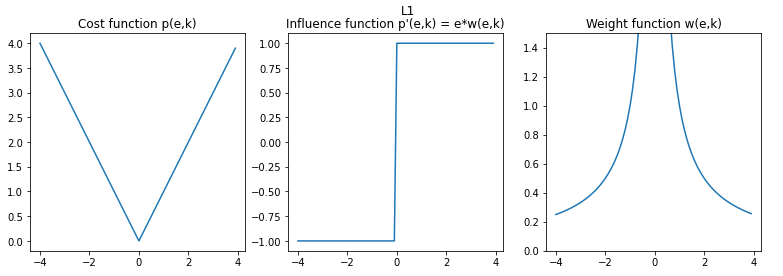

Supplement: Supplemental Information 1 [file peerj-cs-11-2628-s001.zip › code/doc/images/l1_nok.png]

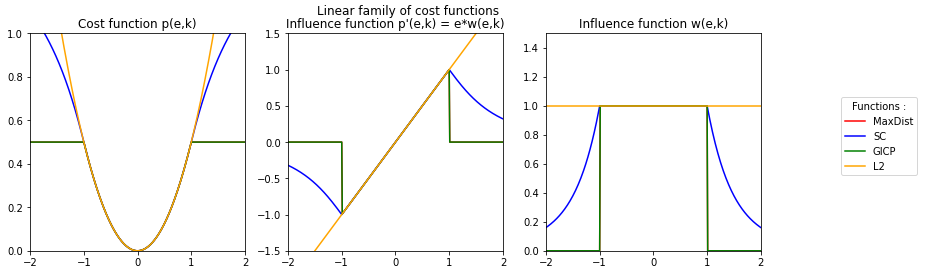

Supplement: Supplemental Information 1 [file peerj-cs-11-2628-s001.zip › code/doc/images/linear_family.png]

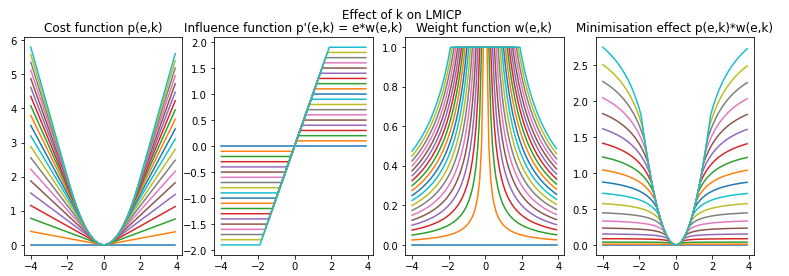

Supplement: Supplemental Information 1 [file peerj-cs-11-2628-s001.zip › code/doc/images/lmicp.png]

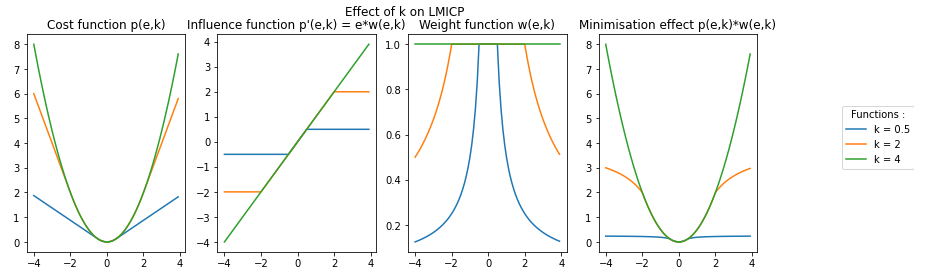

Supplement: Supplemental Information 1 [file peerj-cs-11-2628-s001.zip › code/doc/images/lmicp_v2.png]

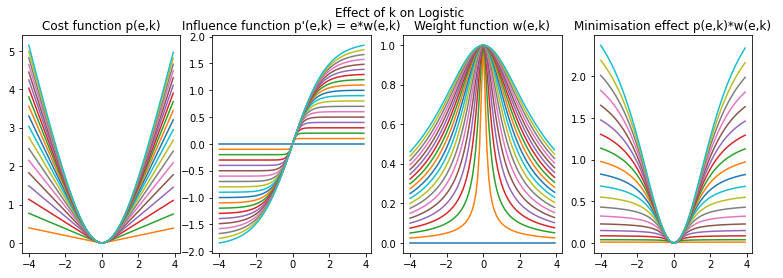

Supplement: Supplemental Information 1 [file peerj-cs-11-2628-s001.zip › code/doc/images/logistic_multik.png]

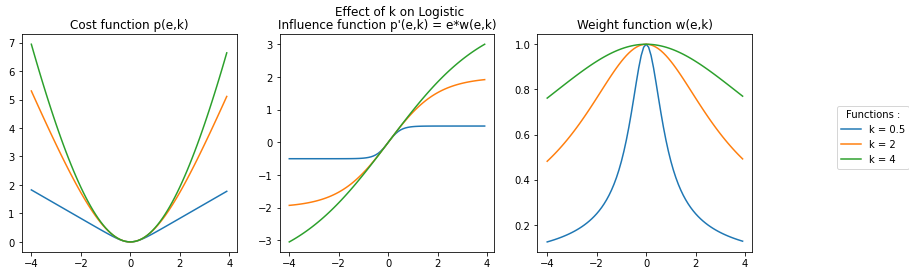

Supplement: Supplemental Information 1 [file peerj-cs-11-2628-s001.zip › code/doc/images/logistic_threek.png]

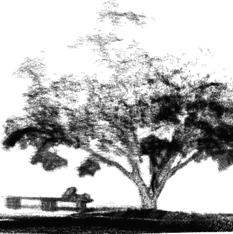

Supplement: Supplemental Information 1 [file peerj-cs-11-2628-s001.zip › code/doc/images/logo.png]

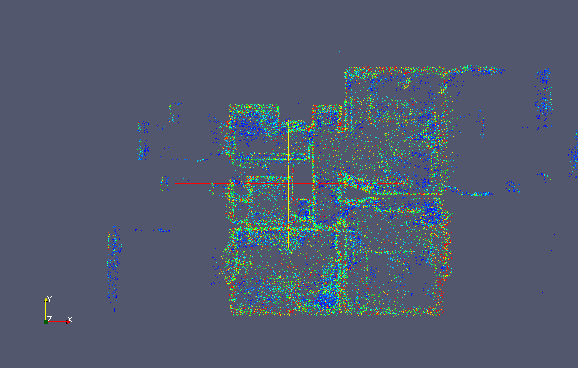

Supplement: Supplemental Information 1 [file peerj-cs-11-2628-s001.zip › code/doc/images/max_dens_after.png]

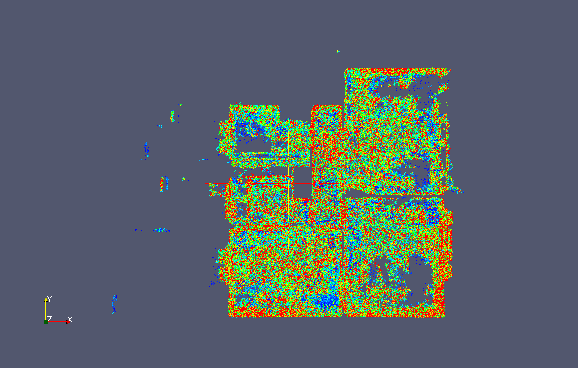

Supplement: Supplemental Information 1 [file peerj-cs-11-2628-s001.zip › code/doc/images/max_dens_before.png]

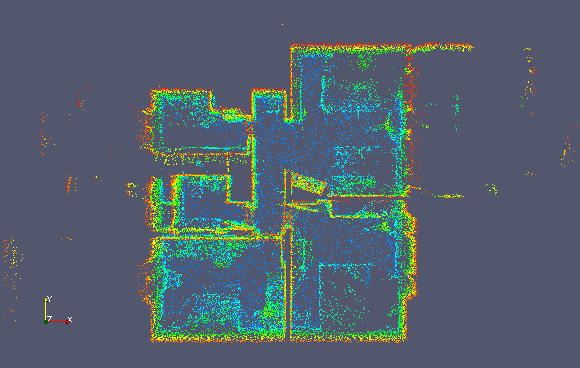

Supplement: Supplemental Information 1 [file peerj-cs-11-2628-s001.zip › code/doc/images/max_num.png]

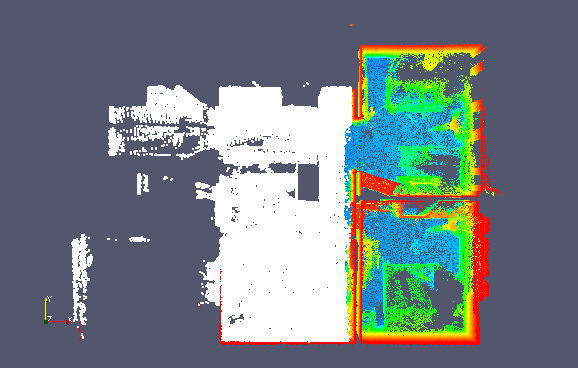

Supplement: Supplemental Information 1 [file peerj-cs-11-2628-s001.zip › code/doc/images/max_quant.png]

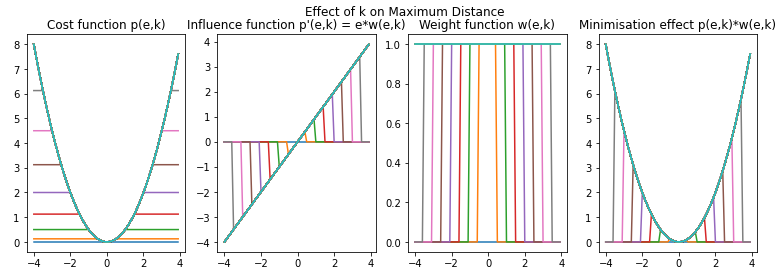

Supplement: Supplemental Information 1 [file peerj-cs-11-2628-s001.zip › code/doc/images/maximumdistance_multik.png]

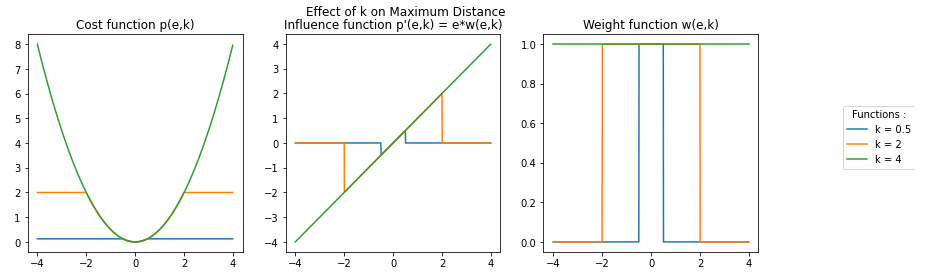

Supplement: Supplemental Information 1 [file peerj-cs-11-2628-s001.zip › code/doc/images/maximumdistance_threek.png]

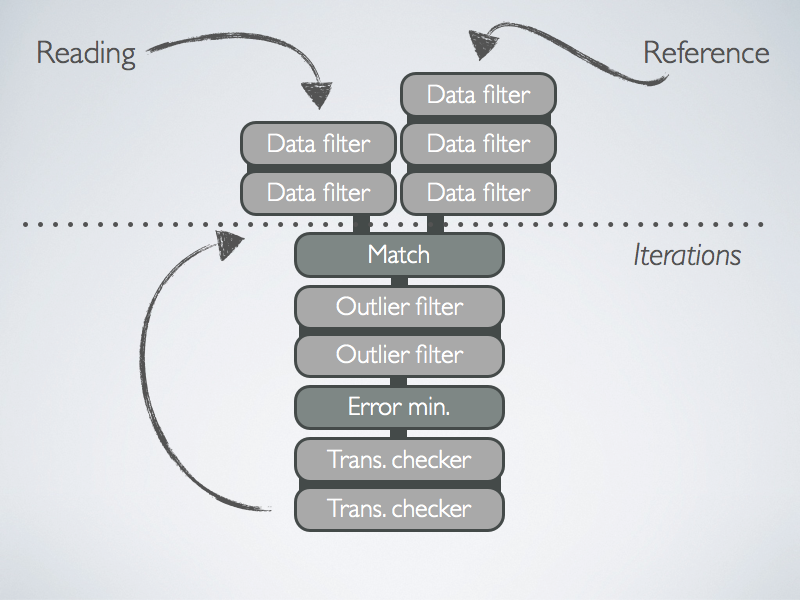

Supplement: Supplemental Information 1 [file peerj-cs-11-2628-s001.zip › code/doc/images/modular_cloud_matcher_icp_chain.png]

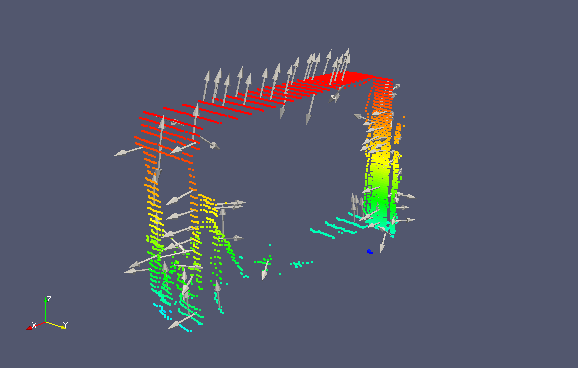

Supplement: Supplemental Information 1 [file peerj-cs-11-2628-s001.zip › code/doc/images/norm.png]
